# Matrix metalloproteinase-1 expression in breast carcinoma: a marker for unfavorable prognosis

**DOI:** 10.18632/oncotarget.20557

**Published:** 2017-08-24

**Authors:** Ji Wang, Chenyang Ye, Demin Lu, Yongxia Chen, Yunlu Jia, Xiaogang Ying, Hanchu Xiong, Wenhe Zhao, Jichun Zhou, Linbo Wang

**Affiliations:** ^1^ Department of Surgical Oncology, Sir Run Run Shaw Hospital, College of Medicine, Zhejiang University, Hangzhou, Zhejiang, 310016, China; ^2^ Biomedical Research Center and Key Laboratory of Biotherapy of Zhejiang Province, Hangzhou, Zhejiang, 310016, China; ^3^ Cancer Institute, Key Laboratory of Cancer Prevention and Intervention, China National Ministry of Education, Second Affiliated Hospital, School of Medicine, Zhejiang University, Hangzhou, Zhejiang, 310009, China; ^4^ Department of Medical Oncology, Second Affiliated Hospital, School of Medicine, Zhejiang University, Hangzhou, Zhejiang, 310009, China

**Keywords:** MMP1, breast cancer, immunohistochemistry, prognosis, overall survival

## Abstract

Matrix metalloproteinase-1 (MMP1) is a member of the matrix metalloproteinases family, and its aberrant expression is implicated in tumor invasion and metastasis. However, the relationship between MMP1 abnormal expression and clinical outcome in breast cancer patients remains to be elucidated. To address this issue, we conducted immunohistochemistry in breast cancer and adjacent normal tissues, and mined the transcriptional and survival data of MMP1 in breast cancer patients through Oncomine, Kaplan-Meier Plotter, bc-GenExMiner, COSMIC and cBioPortal databases. First, we found that both protein and mRNA levels of MMP1 expression were significantly higher in breast cancer tissues. Second, high MMP1 mRNA expression correlated with worse overall survival among grade II (HR = 1.75; p = 0.011), nodal-negative (HR = 2.00; p = 0.00028), ER-positive (HR = 1.61; p = 0.00027) and HER2-negative (HR = 3.17; p = 0.029) patients with breast cancer by using Kaplan-Meier plotter database. Third, the overexpression of MMP1 was associated with unfavorable survival results including overall survival (HR = 1.6; p = 1.6e-05), relapse free survival (HR = 1.78; p < 1e-16) and distant metastasis free survival (HR = 1.65; p = 5.3e-05) in patients with breast cancer. Taken together, the expression status of MMP1 is a significant prognostic indicator and a potential drug target for breast cancer.

## INTRODUCTION

Breast cancer (BC) is one of the most common cancers and one of the major cause of cancer-related deaths worldwide [1]. BC is known to be a heterogeneous disease of distinct histological and biological subtypes with different pathological, molecular and clinical features. Although current prognostic and predictive biomarkers have markedly improved treatment options for patients with BC, intratumor heterogeneity in BC still complicates the diagnosis and treatment, and influences the clinical outcome. Therefore, more reliable markers are still required to further improve therapeutic strategy for individual patients.

Matrix metalloproteinases (MMPs), a family of enzymes that degrade numerous kinds of extracellular matrix (ECM), were reported to play a key role in the metastatic process of cancer cells [2, 3]. Among all the identified MMPs, matrix metalloproteinase-1 (MMP1), also termed as interstitial collagenase or fibroblast collagenase, is the most ubiquitously expressed interstitial collagenase and specifically breaks down the interstitial collagens I, II, and III. A plenty of studies indicated that MMP1 is implicated in the progression and metastasis of tumor cells [4].

High expression of MMP1 has been identified in various kinds of cancers, and its overexpression has been proved to be correlated with unfavorable clinical outcome in malignancies such as hepatocellular carcinoma [5], gallbladder carcinoma [6], thyroid carcinoma [7], pancreatic carcinoma [8], esophageal cancer [9], gastric cancer [10] and colorectal cancers [11]. However, the relationship between abnormal expression of MMP1 and clinical outcome in BC patients remains unknown. Therefore, we investigated the expression of MMP1 in BC and its relationship with the clinicopathological features and clinical outcomes.

In this study, we conducted immunohistochemistry (IHC) assays to value the protein expression level of MMP1 in BC by using tissue microarray consisting of 107 BC samples, and 36 paired IDBC and adjacent normal breast tissues. The relationships between MMP1 protein expression and clinicopathological features, such as age, nodal involvement, and ER, PR, HER2 status were also analyzed. We used Oncomine and breast cancer gene-expression miner (bc-GenExMiner) databases to assess MMP1 mRNA expression between BC cancer tissues and adjacent normal samples, and the correlations between MMP1 mRNA expression and clinicopathological characteristics. We then probed into the prognostic merit of MMP1 by survival analysis on the Kaplan-Meier Plotter database. Lastly, the genetic alterations and outcomes of cancer patients on cBioPortal online database were assessed.

## RESULTS

### Protein level expression of MMP1 in breast cancer patients

**Figure 1:**
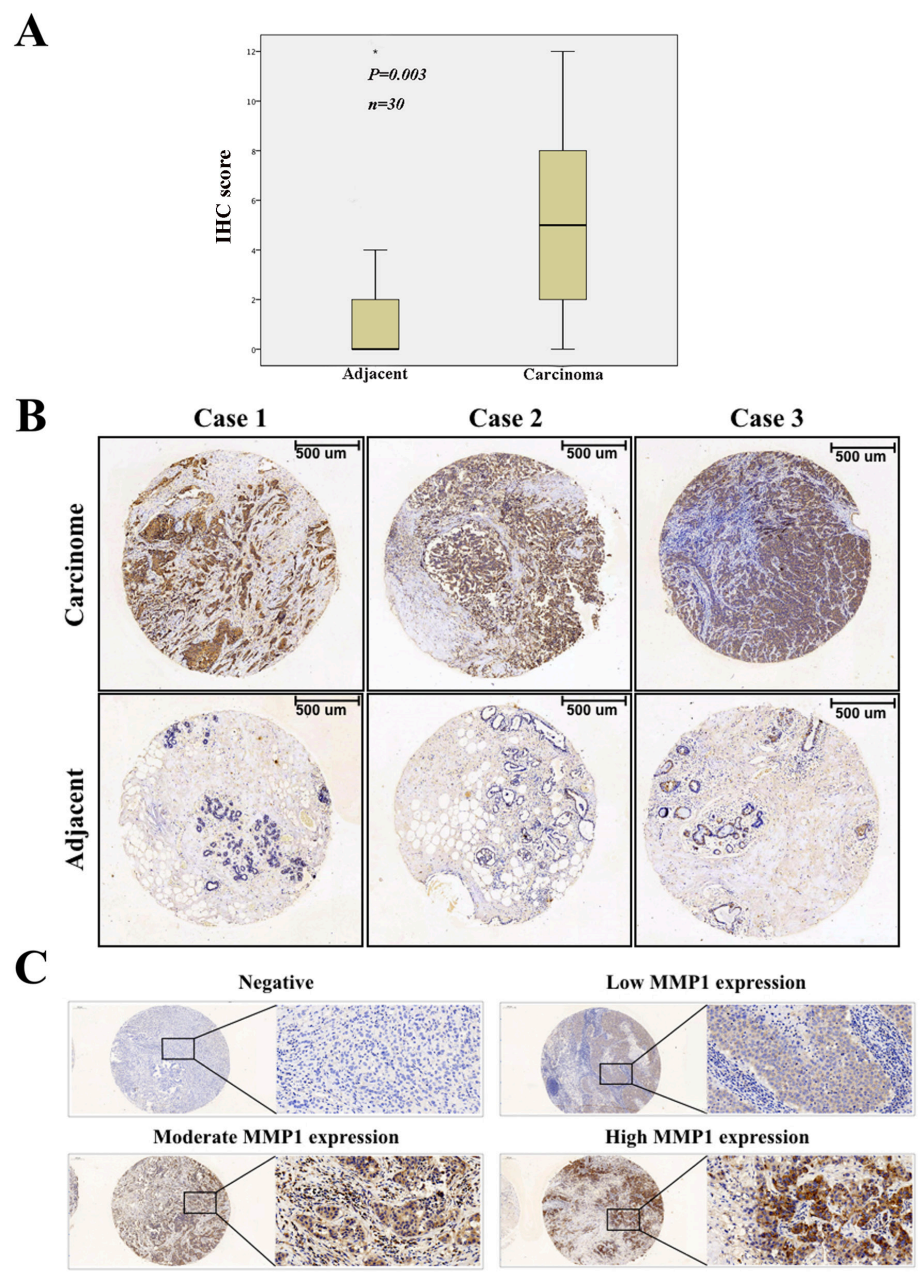
Immunohistochemical staining of MMP1 protein in BC **(A)** Expression level of MMP1 in BC was significantly higher than corresponding controls (p = 0.003). **(B)** Representative images of immunohistochemical staining of MMP1 expression in BC samples and matched adjacent normal tissues. **(C)** Negative, low, moderate and high MMP1 expression staining of BC. Scale bars, 100 μm.

To investigate the protein expression level of MMP1 in BC, we assessed BC tissue samples and matched adjacent normal tissues from 36 human cases by using immunohistochemistry (IHC). IHC analysis indicated that MMP1 expression was significantly elevated in cancerous tissues compared with corresponding normal tissues (p = 0.003) (Figure 1A–1B).

### Relationship of MMP1 protein expression with the clinicopathological characteristics

To better understand the relevance of MMP1 to BC, we divided the 143 BC samples into groups based on the clinicopathological variables and evaluated the differences in MMP1 expression among these groups (Table 1). According to the classification of MMP1 IHC staining (Figure 1C), Positive MMP1 expression was observed to be positively related with the T stage (p = 0.001), while the expression of MMP1 was negatively associated with ER and PR status (p = 0.005 and 0.027, respectively). However, there were no significant association between MMP1 expression and other clinicopathologic factors including age, tumor location, differential grade, lymph node infiltration, and HER2 and triple-negative breast cancer (TNBC) status (p = 0.377, 0.856, 0.394, 0.5, 0.861 and 0.188, respectively).

**Table 1:** Clinicopathological variables and the protein level expression of MMP1 in total BC patients according to the immunohistochemistry analysis

Characteristic	Cases	MMP1 expression level		
		No. of Low expression	No. of High expression	P value
Age (years)				0.377
<50	52	24	28	
>50	90	34	56	
Tumor location				0.856
left	74	26	48	
right	58	22	36	
Differential grade				0.394
I	4	1	3	
II	96	43	53	
III	36	12	24	
Lymph node infiltrated				0.5
Yes	69	27	42	
No	73	33	40	
T factor				0.001^*^
T1/2	112	55	57	
T3/4	27	4	23	
ER status				0.005^*^
Positive	87	44	43	
Negative	54	14	40	
PR status				0.027^*^
Positive	70	36	34	
Negative	72	23	49	
HER-2 status				0.861
Positive	87	36	51	
Negative	53	21	32	
TNBC status				0.188
TNBC	17	4	13	
Non-TNBC	124	53	71	

### Transcription levels of MMP1 in breast cancer patients

**Figure 2:**
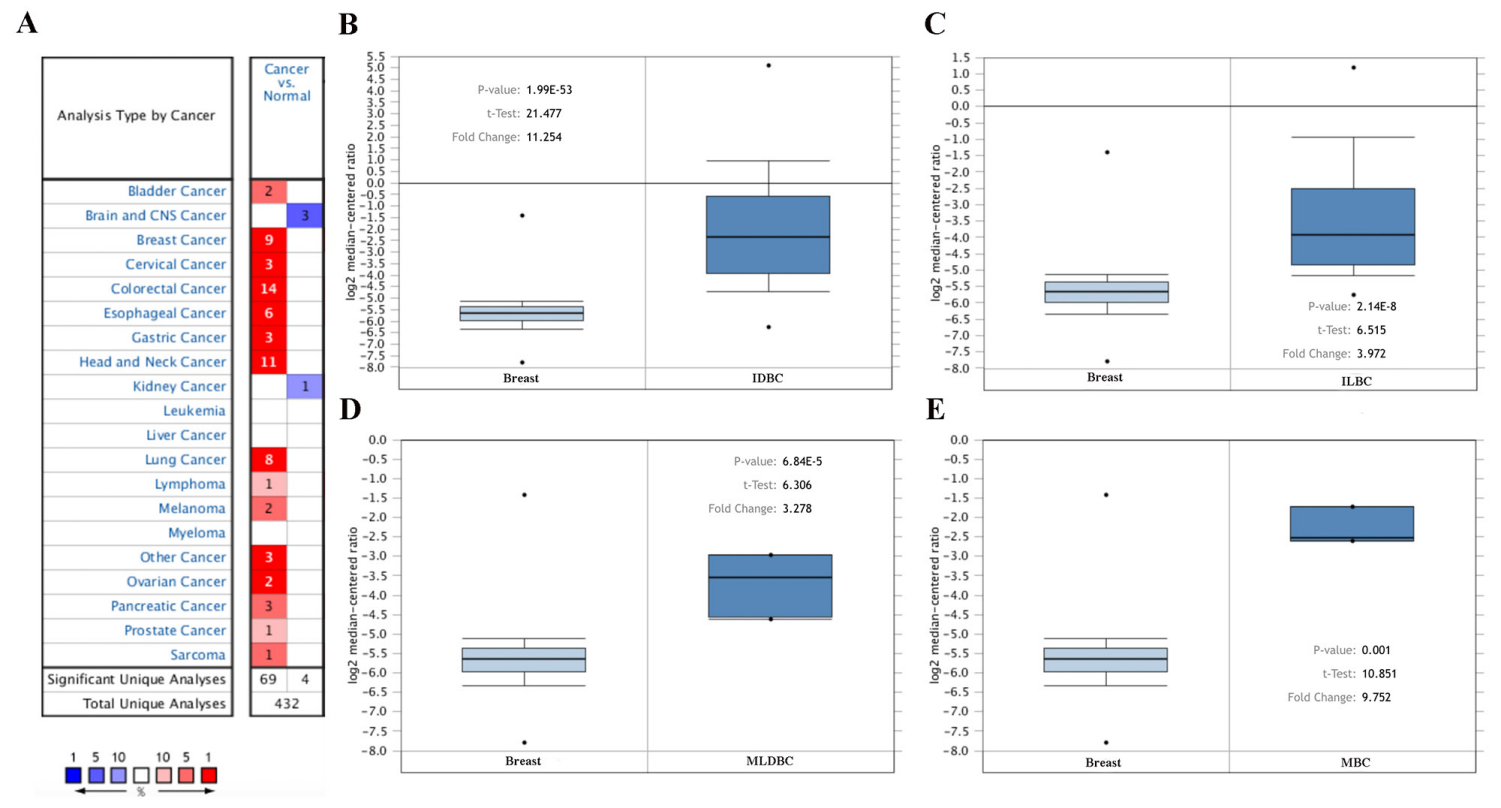
MMP1 mRNA expression in malignant tumors (Oncomine database) **(A)** The graph is a representation of the datasets with statistically significant mRNA overexpression (red) or reduced expression (blue) of MMP1 gene (cancer vs normal). Cell color was determined by the best gene rank percentile for the analyses within the cell, and the gene rank was analyzed by percentile of target gene in the top of all genes measured in each research. **(B)** Comparison of MMP1 mRNA expression between normal breast tissue and IDBC. **(C)** Comparison between normal breast tissue and ILBC. **(D)** Comparison between normal breast tissue and MLDBC. **(E)** Comparison between normal breast tissue and MBC.

The mRNA expression feature of MMP1 was presented by using the SAGE Digital Gene Expression Display. Higher level of MMP1 mRNA in breast cancer tissue was identified compared with matched normal tissues (Supplementary Figure 1). Based on the Oncomine database, we discovered that MMP1 expression was significantly elevated in breast cancer samples compared with normal samples in nine datasets (Figure 2A). In TCGA’s dataset, the transcription levels of MMP1 in different types of breast cancer were higher than normal tissues, including invasive ductal breast carcinoma (IDBC) with fold change = 11.254, invasive lobular breast carcinoma (ILBC) with fold change =3.972, mixed lobular and ductal breast carcinoma (MLDBC) with fold change = 3.278, and male breast carcinoma (MBC) with fold change = 9.752. (Figure 2B–2D).

### The relationship between mRNA levels of MMP1 and clinicopathological parameters of breast cancer patients

**Figure 3:**
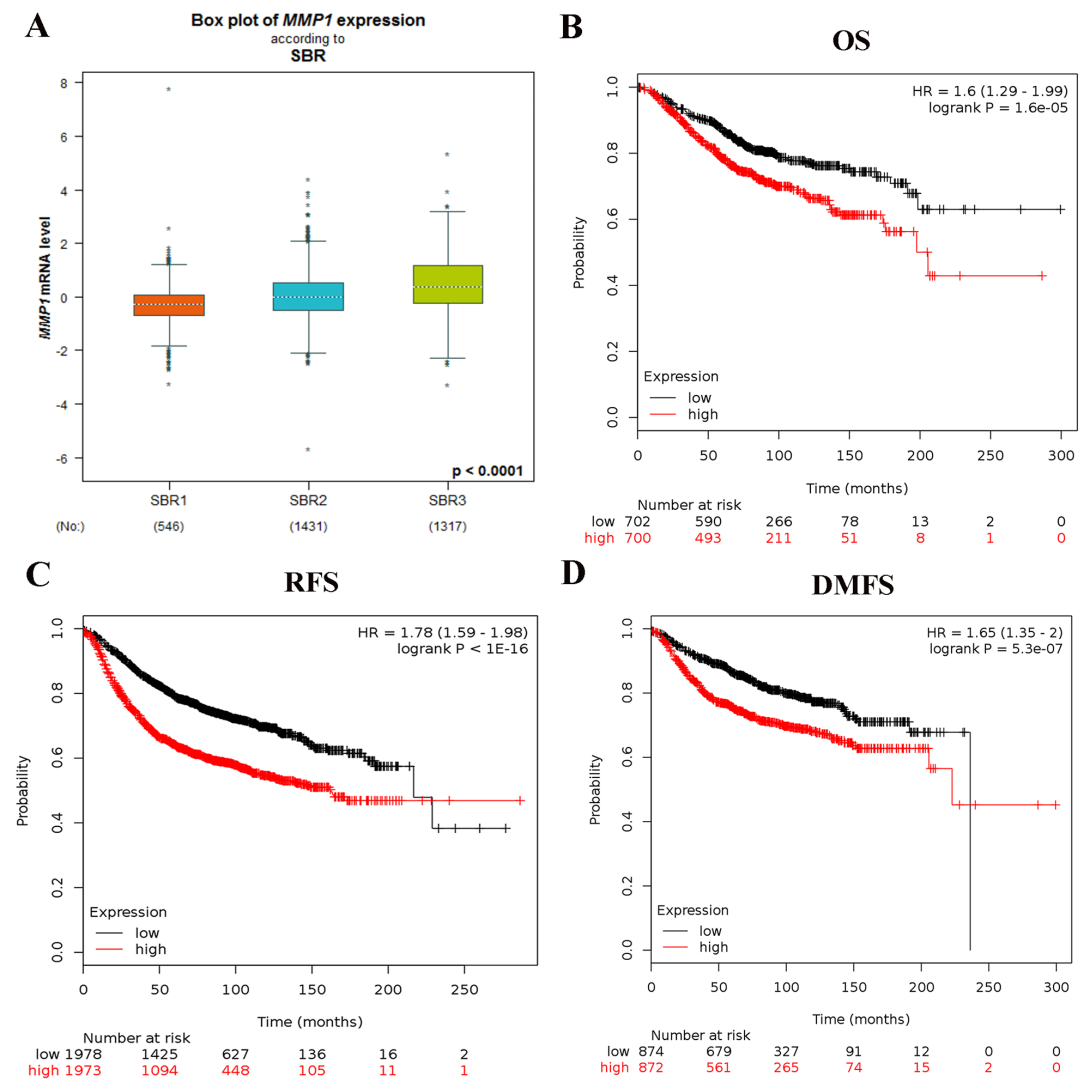
**(A)** The relationship between mRNA expression of MMP1 and SBR. The prognostic value of mRNA level of MMP1 in BC patients for **(B)** OS, **(C)** FP, and **(D)** DMFS in Kaplan-Meier plotter.

In bc-GenExMiner, the Welch’s test was performed to compare the mRNA expression of MMP1 among groups of patients in light of different clinicopathological parameters (Table 2). For age criterion, no significant differences between ≤ 51 y and > 51 y groups were found. BC patients with positive nodal status showed higher MMP1 expression than negative-nodal patients. ER and PR status were found to be negatively correlated with MMP1 expression. In BC patients with HER2 overexpression, the transcription level of MMP1 was significantly increased compared with HER2-negative groups. TNBC is a special type of BC, with ER (-), PR (-) and HER2 (-). We identified the expression level of MMP1 was significantly upregulated in non-TNBC patients. In Scarff Bloom & Richardson grade status (SBR) criterion, more advanced SBR grade was associated with the higher mRNA level of MMP1 (Figure 3A).

**Table 2:** The relationship between mRNA expression of MMP1 and clinicopathological parameters of breast cancer (from the breast cancer gene-expression miner v4.0).

Subgroup analysis	Cases	mRNA	p value
Age			
≤ 51	1392	-	0.2293
> 51	2210	-	
Nodal status			
negative	2493	-	0.0040^*^
positive	1562	↑	
ER (IHC)			
negative	1446	↑	< 0.0001^*^
positive	3766	-	
PR (IHC)			
negative	804	↑	< 0.0001^*^
positive	1249	-	
HER2 (IHC)			
negative	1409	-	= 0.0001^*^
positive	201	↑	
Triple-negative Status			
Not	374	↑	< 0.0001^*^
TNBC	3857	-	

### The prognostic merit of MMP1 in breast cancer

The prognostic merit of MMP1 mRNA expression was analyzed by using the online Kaplan-Meier Plotter database. The Kaplan-Meier curve and log-rank test analyses showed that increased levels of MMP1 were significantly correlated with worse overall survival (OS) (HR = 1.6; 95% CI: 1.29 - 1.99, p = 1.6e-05), relapse free survival (RFS) (HR = 1.78; 95% CI: 1.59 - 1.98, p < 1E-16) and distant metastasis free survival (DMFS) (HR = 1.65; 95% CI: 1.35 - 2, p = 5.3e-05) for BC patients (Figure 3B–3D).

The relationship of MMP1 mRNA expression with lymph node status of BC patients was then investigated (Table 3). Upregulated MMP1 was significantly related to worse OS in negative-nodal patients (HR = 2.00, 95% CI: 1.36-2.92, p = 0.00028), while not correlated to OS in positive-nodal patients (HR = 1.13; 95% CI: 0.76-1.66, p = 0.55).

**Table 3:** Correlation of MMP1 mRNA expression and clinical survival of breast cancer patients with different clinicopathological factors (from the Kaplan-Meier Plotter)

Subgroup analysis	Patient number	HR (95% CI)	P value
Nodal status			
negative	594	2 (1.36-2.92)	0.00028^*^
positive	313	1.13 (0.76-1.66)	0.55
Grade			
I	161	1.94 (0.76-4.94)	0.16
II	387	1.75 (1.13-2.7)	0.011^*^
III	503	1.1 (0.8-1.53)	0.56
ER			
negative	358	1.1 (0.75-1.63)	0.62
positive	1044	1.61 (1.24-2.09)	0.00027^*^
PR			
negative	89	1.01 (0.4-2.55)	0.98
positive	83	1.34 (0.35-5.09)	0.67
HER2			
negative	130	3.17 (1.06-9.48)	0.029^*^
positive	129	1.25 (0.62-2.5)	0.53

HR: hazard ratio; CI: confidence interval.

We then examined the correlation of MMP1 mRNA expression and clinical outcome in BC patients with diverse ER, PR, and HER2 status (Table 3). MMP1 overexpression was associated with worse OS in ER-positive BC patients (HR = 1.61; 95% CI: 1.24 - 2.09, p = 0.00027), but not associated with OS of ER-negative patients (HR = 1.1; 95% CI: 0.75 - 1.63, p = 0.62). MMP1 mRNA expression was found not linked to OS in both PR-positive and PR-negative BC patients (HR = 1.34; 95% CI: 0.35-5.09, p = 0. 67; HR = 1.01; 95% CI: 0.4 - 2.55, p = 0.98). High MMP1 mRNA expression was correlated to worse OS in HER2-negative BC patients (HR = 3.17; 95% CI: 1.06 - 9.48, p = 0.029), but not related with OS of HER2-positive patients (HR = 1.25; 95% CI: 0.62 - 2.5, p = 0.53).

**Figure 4:**
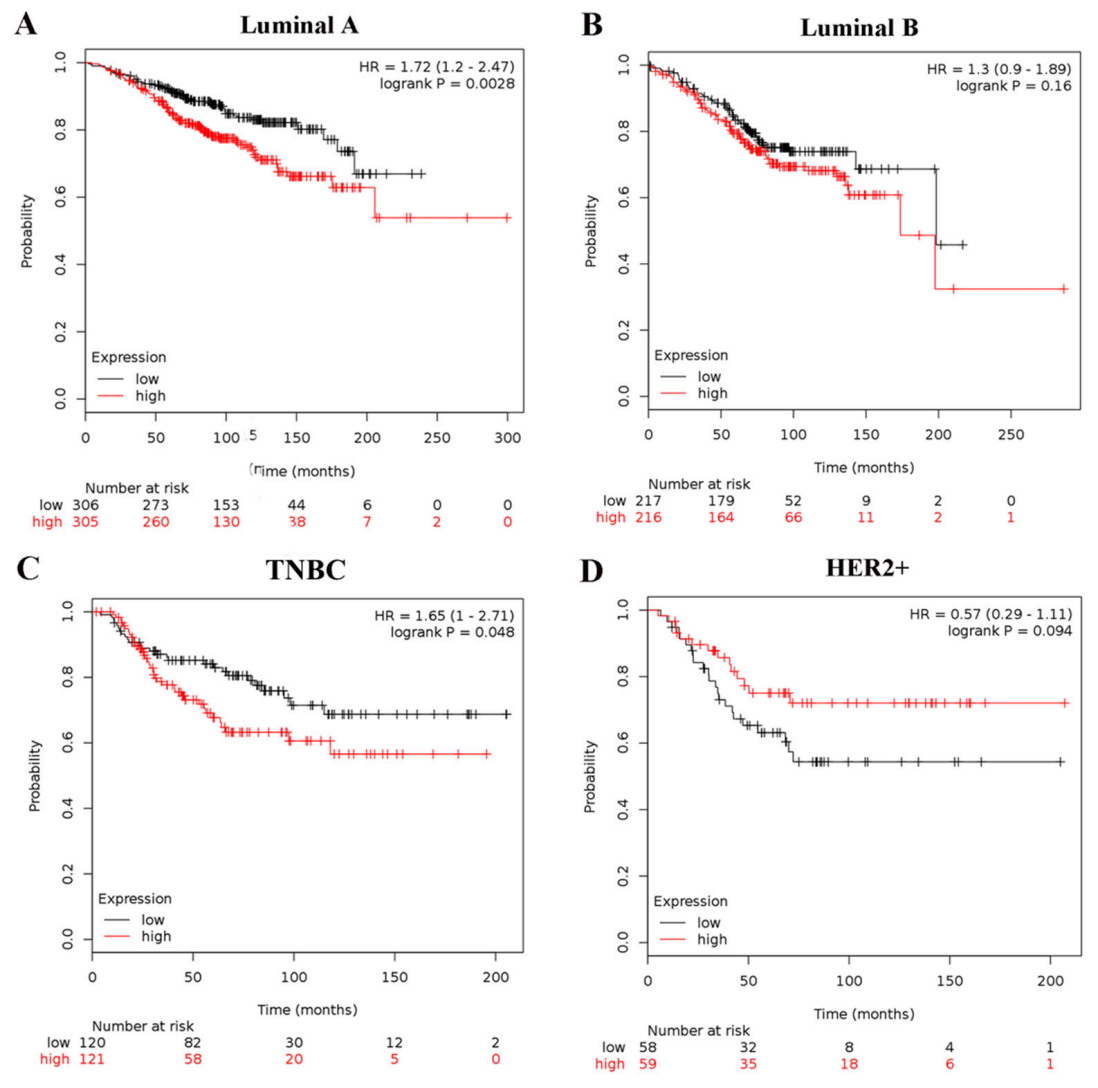
Association of MMP1 with different molecular subtypes of BC patients OS curves are plotted for patients of **(A)** luminal A, **(B)** luminal B; **(C)** TNBC subtype; **(D)** HER2-positive.

To further probe into the correlation of MMP1 mRNA expression and survival, BC patients with diverse differentiation grades and molecular subtypes were also investigated. High MMP1 mRNA expression in grade II differentiated was associated with poor OS of BC patients (HR = 1.75; 95% CI: 1.13-2.7, p = 0.011). But, grade I and III differentiated BC patients showed no correlation with OS of MMP1 mRNA expression (HR=1.94; 95% CI: 0.76-4.94, p = 0.16; HR = 1.1; 95% CI: 0.8-1.53, p = 0.56). As for the molecular breast cancer subtype, upregulated MMP1 was significantly related to worse OS in luminal A and TNBC subtype patients (HR = 1.72; 95% CI, 1.2 - 2.47, p = 0.0028; HR = 1.65; 95% CI: 1-2.71, p = 0.048), while not correlated to OS in luminal B and HER2-positive subtypes of BC patients (HR = 1.3; 95% CI: 0.9-1.89, p = 0.16; HR = 0.57; 95% CI: 0.29-1.11, p = 0.094) (Figure 4A–4D).

### The impact of alterations in MMP1 on the clinical survival

**Figure 5:**
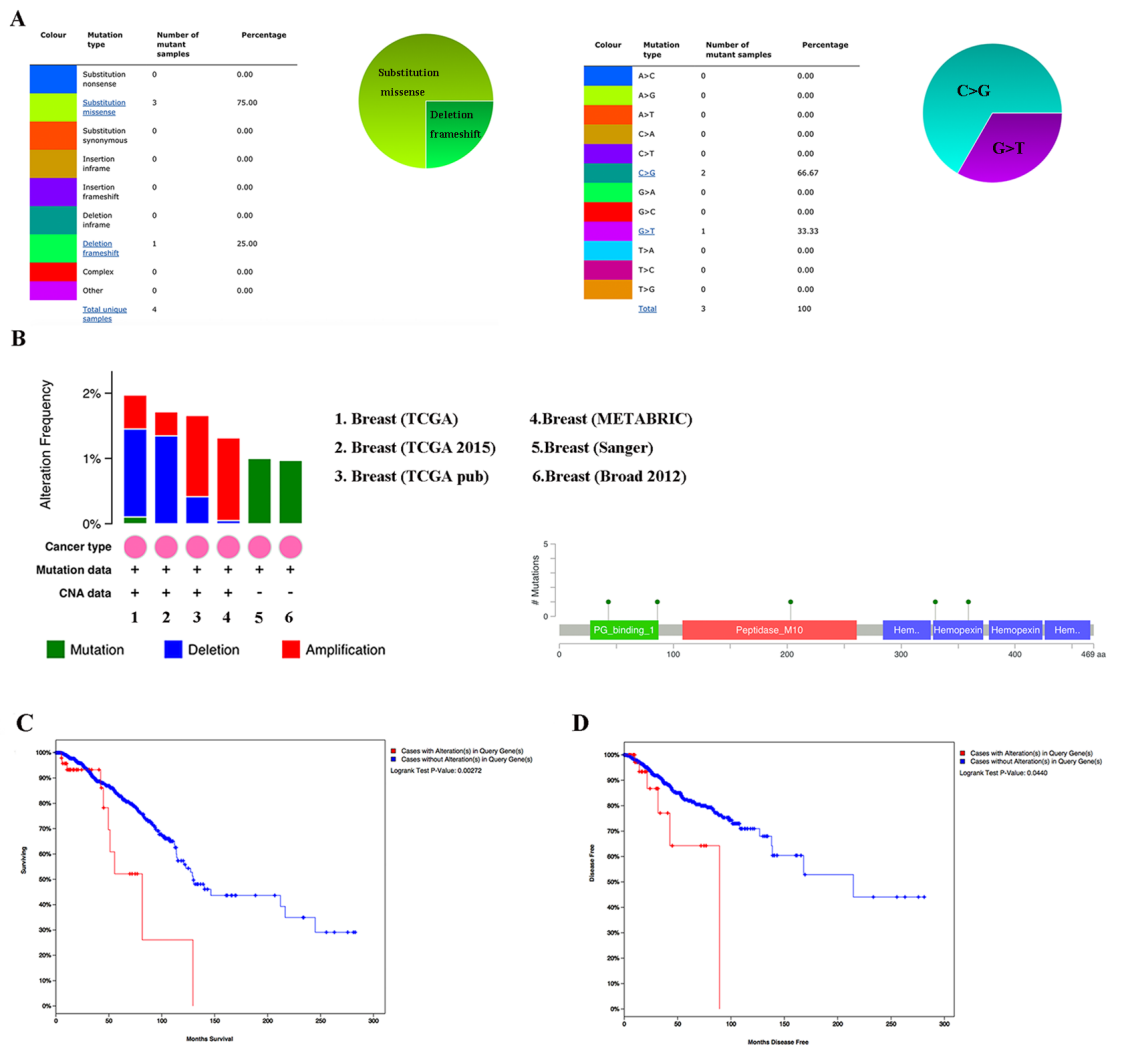
MMP1 genes expression and mutation analysis in breast invasive carcinoma (COSMIC and cBioPortal) **(A)** Pie-chart showed the percentage of the mutation type of MMP1 in BC according to COSMIC database. **(B)** Oncoprint in cBioPortal represented the proportion and distribution of samples with alterations in MMP1 gene. **(C)** Kaplan-Meier plots comparing OS in cases with/without MMP1 gene alterations. **(D)** Kaplan-Meier plots comparing disease free survival (DFS) in cases with/without MMP1 gene alterations.

By using COSMIC, the pie chart described the mutations information including substitution nonsense, missense, synonymous, deletion frame and insertion frame shift. Substitution missense rate was 75.00% and deletion frameshift rate was 25.00% of mutant samples of BC. BC had 66.67% C > G and 33.33% G > T mutation in MMP1 coding strand (Figure 5A). Alteration frequency of MMP1 mutation in BC was analyzed by using cBioPortal. Less than 2% mutation in the patients with BC was observed (Figure 5B). After analyzed by Kaplan-Meier plot and log-rank test, the alterations in MMP1 were correlated with worse OS (p=0.00272) and DFS (p=0.044) in BC patients with/ without MMP1 alterations (Figure 5C–5D).

## DISCUSSION

BC is one of the most common malignant tumors among women worldwide [1]. Although remarkable improvements in early detection and personalized therapeutics have decreased mortality of BC in recent years, the prevention and treatment of BC are still considerable public health concerns [12]. Therefore, novel prognostic indicators are necessary to further improve the prognosis of BC patients.

MMP1 is a proteolytic enzyme which degrades ECM, and its upregulated expression status has been detected among several kinds of malignant tumors [6, 13, 14]. Animal researches revealed that overexpression status of MMP1 played a part in initiating mammary tumorigenesis through breaking down stroma and disseminating growth factors and mitogens for epithelial cells [15]. ECM degradation caused by MMP1 was proved to perturb the interactions between cell-cell and cell-ECM and separate cells from ECM, resulting in decreased apoptosis, enhanced cell division, and tumorigenesis [16]. Abnormal expression of MMP1 was identified in several types of malignant cancers [11, 17], but its expression status and prognostic merit in BC still remains unclear. Given the important role of MMP1 both in mechanism of cancer carcinogenesis and clinical application, we carried out this research to assess the clinical and prognostic importance of MMP1 in BC.

In IHC analysis, we identified that MMP1 protein expression was significantly upregulated in BC cancerous tissues than corresponding normal tissues. We also analyzed the relationship between MMP1 protein expression, tumor size, lymph node metastasis, differential grade, and ER, PR, HER2 status. We detected that MMP1 overexpression in BC significantly correlated with tumor size (p=0.001), ER (p=0.005) and PR (p=0.027) status. We then used Oncomine database to elucidate MMP1 mRNA expression in different types of cancers and different subtypes of BC. The results indicated that the MMP1 expression was significantly higher in tumor samples compared with normal samples at mRNA level. Thus, overexpression status of MMP1 in BC cancerous tissues compared with adjacent normal ones was verified at both mRNA and protein levels. And advanced SBR grade was positively correlated with higher mRNA level of MMP1.

Then, KM plotter was conducted to evaluate the correlation of MMP1 mRNA to clinical outcome. We concluded that the mRNA expression of MMP1 was associated to unfavorable OS, PFS and DMFS for all BC patients, which revealed that MMP1 mRNA expression may serve as an indicator for prevention and prognosis of BC. Regarding to nodal involvement and differential grade of patients with BC, high expression of MMP1 contributed to worse OS in grade II and nodal-negative BC patients, whereas not associated to OS of grade I and III, and nodal-positive patients. As for the BC patients with different receptor subtypes, overexpression of MMP1 mRNA level was correlated to worse OS in ER-positive and HER2-negative BC patients, while not associated with ER-negative, HER2-positive patients. This result was consistent with the analysis about luminal A subtype (ER-positive and HER2-negative). HER2 is a member of epidermal growth factor receptor family and was identified to be overexpressed in 30% breast cancers [18, 19]. HER2 played vital roles in the development and progression of some aggressive types of BC, and was found to be related with poor clinical outcomes [19, 20]. Thus, more researches are warranted to find out whether MMP1 protein influences the HER2 status or they perform competitively or collectively toward the prognosis in the BC setting. In light of our analysis, high expression of MMP1 mRNA was correlated with worse OS among grade II, nodal-negative, ER-positive and HER2-negative patients with BC.

Genetic polymorphisms are very important for malignant tumors, and can also impose vital impact as independent prognostic markers on therapeutic strategies for cancer patients. However, the role of MMP1 polymorphism remains controversial. Although one study revealed that MMP1 polymorphisms was found not correlated with BC risk in the assessment among 3,016 BC cases and 3,007 controls [21], other studies indicated that MMP1 single nucleotide polymorphisms (SNPs) were related to BC clinical survival [22]. MMP1 2G SNP was significantly correlated with BC progression and could be used as a prognostic indicator [23, 24]. Based on current literature, MMP1 polymorphisms are promising biomarkers, and further researches should be carried out to figure out the prognostic role of MMP1 polymorphisms in BC patients.

In summary, results of our research involve some important implications. First, high MMP1 expressions at both mRNA and protein levels were found in BC cancer tissues compared with corresponding normal samples. Second, overexpression of MMP1 mRNA was related with unfavorable OS among grade II, nodal-negative, ER-positive and HER2-negative patients with BC. Third, the elevated expression of MMP1 was related with unfavorable survival outcome in BC patients, suggesting that MMP1 could serve as a prognostic indicator. Lastly, although further researches are required, MMP1 genetic polymorphisms are promising biomarkers for prognosis of BC patients.

## MATERIALS AND METHODS

### Patients and samples

Tissue microarray was provided by Servicebio (Wuhan, China). There were 179 samples included in the microarray with 107 invasive ductal breast cancer (IDBC) tumor tissues and another 36 paired IDBC and adjacent nonmalignant breast tissues.

### Immunohistochemistry

Breast cancer tissue microarray (TMA) was heated, deparaffinized and were treated with citrate antigen repair buffer (pH 6.0) to antigen repair, with 3% hydrogen peroxide to block endogenous peroxidase activity, with 3% BSA to serum blocking. Tissue microarray was incubated with MMP1 primary antibody (1:200; polyclonal antibody; HuaAn Bio-Technology) and coordinate secondary antibody. Staining was displayed with DAKO DBA solution. Harris hematoxylin was used to re-stain the nucleus and TMA was dehydrated by alcohol.

The stained TMA was scanned using the Pannoramic Midi and analyzed using the Pannoramic Viewer (3D Histech) and Quant center. The software automatically identified and set all dark brown on the tissue slice =3, brown yellow=2, light yellow=1, blue nucleus=0, and the extent of stained cells (0-5%=0; 5-25%=1; 26-50%=2; 51-75%=3 and 76-100%=4). The final score was determined by multiplying the intensity score and the score for the extent of stained cells, generating a score that ranged from 0 to 12. The staining results were categorized into negative (score 0; −), low (score 1–4; +), moderate (score 5–8; ++), and high (score 9–12; +++). The results were evaluated by two independent pathologists.

### Oncomine database analysis

The online cancer microarray database, Oncomine (www.oncomine.org) [25] was used to assess the transcription levels of MMP1 in breast cancer (BC) specimens compared with that in normal controls by Students’ *t*-test. The threshold of *p* value, fold change and gene rank were 0.01, 2 and top 10%, respectively.

### Serial analysis of gene expression (SAGE)

All available published SAGE data were utilized for analysis of MMP1 gene expression between cancerous and normal tissues. Digital MMP1 gene expression profiles were evaluated by using SAGE Genie tools (http://www.ncbi.nlm.nih.gov/SAGE/) [26].

### Breast cancer gene-expression miner v4.0

Breast Cancer Gene-Expression Miner v4.0 (bc-GenExMiner v4.0) consisted 36 annotated genomic datasets and three statistical mining functions [27, 28]. The expression module was added on 2016/03/30, comparing the expression of a target gene based on clinical criteria, such as age, hormonal receptors, nodal status, and so on. The prognostic module evaluated the prognostic merit of candidate genes in human BC and provided potential prognostic indicators for BC. The correlation module computed the association between genes or identified clusters of correlated co-expressed genes located in the same chromosomal region.

### The Kaplan-Meier Plotter

The prognostic merit of MMP1 mRNA expression was appraised by an online database, Kaplan-Meier Plotter (www.kmplot.com) [29], which included gene expression data and survival information of clinical BC patients from Gene Expression Omnibus database. To analyze the overall survival (OS), relapse free survival (RFS) and distant metastasis free survival (DMFS) of patients with BC, patient samples were split into two groups by median expression (high vs. low expression) and assessed by a Kaplan-Meier survival plot, with the hazard ratio (HR) with 95% confidence intervals (CI) and log-rank p value. The Affymetrix ID is valid: 204475_s_at (MMP1).

### COSMIC analysis for mutations

Catalogue of Somatic Mutations in Cancer (COSMIC) database (http://www.sanger.ac.uk/cosmic/) was utilized for assessment of MMP1 mutations [30]. Pie charts were generated for a distribution overview and substitutions on the coding strand in BC.

### TCGA data and cBioPortal

The Cancer Genome Atlas had both sequencing and pathological data on 30 different cancers [31]. The database query was based on mutation and altered expression of the MMP1 in invasive breast carcinoma (TCGA, Cell 2015; TCGA, Nature 2012; TCGA, Provisional; METABRIC, Nature 2012& Nat Commun 2016; Sanger, Nature 2012; Broad 2012). The breast invasive carcinoma (TCGA, Provisional) dataset including data from 1105 samples with pathology reports was selected for further analyses of MMP1 using cBioPortal (www.cbioportal.org) [32, 33]. The genomic profiles included mutations, copy-number variance (CNV) from GISTIC, mRNA expression z-scores (RNA Seq V2 RSEM) and protein expression z-scores (RPPA). OS and DFS were calculated according to the cBioPortal’s online instruction.

### Statistical analysis

Statistical analysis was performed using SPSS 23.0 software. The expression of MMP1 in BC samples and corresponding adjacent tissues, as well as differential grade were compared by using the Wilcoxon test. Associations between MMP1 expression in BC tissues and clinicopathological features were assessed by chi-square test. All statistical tests were two-sided, and statistical significance was defined as P value less than 0.05.

## SUPPLEMENTARY MATERIALS FIGURE


