# Supplementary material for: Matrix metalloproteinase-1 expression in breast carcinoma: a marker for unfavorable prognosis
Source: Oncotarget. 2017 Aug 24;8(53):91379–90. doi: 10.18632/oncotarget.20557 (PMC5710931; doi:10.18632/oncotarget.20557)
Supplement: Supplementary file 1 [file oncotarget-08-91379-s001.pdf]

Matrix metalloproteinase-1 expression in breast carcinoma: a marker for unfavorable prognosis

SUPPLEMENTARY MATERIALS

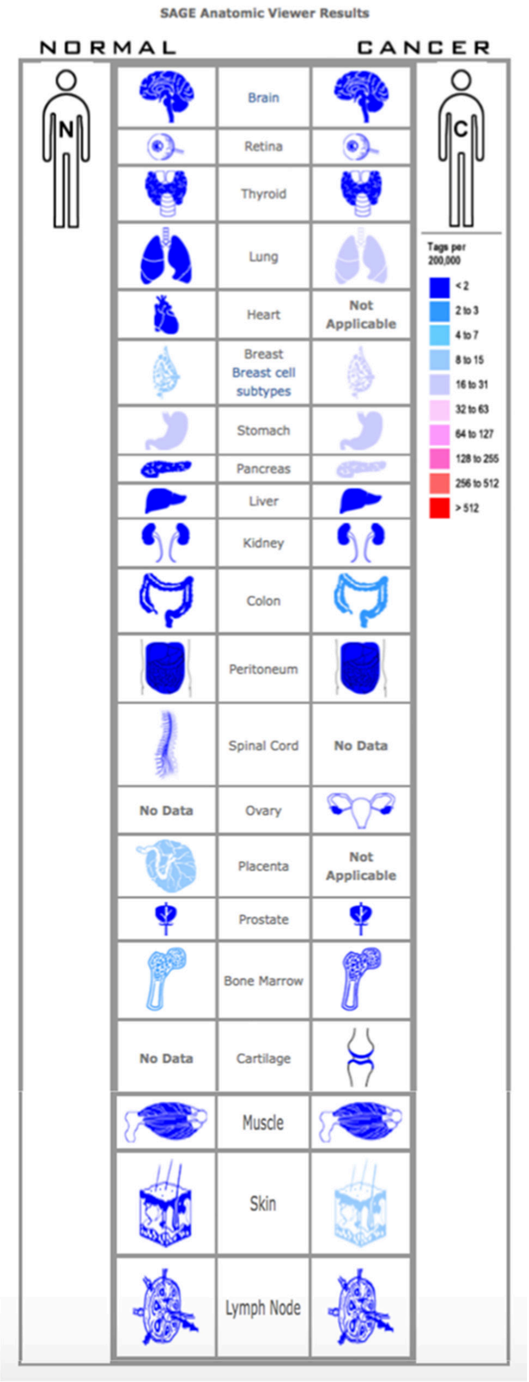

Supplementary Figure 1: Expression profile for MMP1 in common human cancers presented by the SAGE DGED.
